# Regulatory B cells induced by pancreatic cancer cell-derived interleukin-18 promote immune tolerance via the PD-1/PD-L1 pathway

**DOI:** 10.18632/oncotarget.22976

**Published:** 2017-12-07

**Authors:** Yan Zhao, Ming Shen, Yecheng Feng, Ruizhi He, Xiaodong Xu, Yu Xie, Xiuhui Shi, Min Zhou, Shutao Pan, Min Wang, Xingjun Guo, Renyi Qin

**Affiliations:** ^1^ Department of Biliary-Pancreatic Surgery, Affiliated Tongji Hospital, Tongji Medical College, Huazhong University of Science and Technology, Wuhan, China

**Keywords:** pancreatic cancer, interleukin-18, regulatory B cells, immune tolerance, PD-1/PD-L1 pathway

## Abstract

Dysregulation of regulatory B cells (Bregs), a type of immunosuppressive lymphocyte, are associated with development of autoimmune diseases and cancers. Bregs produce immune tolerance-inducing cell surface molecules and tolerogenic cytokines (interleukin [IL]-10 and transforming growth factor-beta). We previously showed that levels of the inflammatory cytokine IL-18 were increased in patients with pancreatic cancer. In the present study study, we found that pancreatic cancer cell-derived IL-18 increases Breg-induced immunosuppression. IL-18 also promoted B-cell proliferation and IL-10 expression *in vivo* and *in vitro*. In addition, IL-18 upregulated membrane PD-1 in B cells and inhibited the antibody-dependent cellular cytotoxicity of Tc cells and natural killer cells. Finally, the combination of a natural IL-18 inhibitor (IL-18BP) and a PD-1/PD-L1 inhibitor suppressed tumor growth and metastasis in a murine pancreatic cancer model. Our results show that IL-18 and PD-1/PD-L1 could be therapeutic targets in pancreatic cancer.

## INTRODUCTION

Pancreatic cancer (PC) is one of the most lethal malignancies. Mutation and alteration of driver genes, such as KRAS, CDKN2A, TP53, and SMAD4, are critical events in pancreatic tumorigenesis [1]. Treatment of PC includes surgery, chemotherapy, radiation therapy, and palliative care. However, despite significant research and therapeutic development efforts, little improvement in survival has been achieved over the past few decades.

Because immune disorders are a major cause of tumorigenesis, we decided to explore immune mechanisms related to pancreatic tumorigenesis. Tumor cells can gain a selective survival advantage by enhancing immune tolerance either directly or indirectly, thus resulting in their proliferation [2]. Therefore, immunotherapy has emerged as a therapeutic option for some cancers.

Interleukin (IL)-18 plays a critical role in inflammation and immune responses. Early evidence indicated that IL-18 had anticancer effects as a result of its ability to induce interferon-gamma (IFN-γ) production in T cells and natural killer (NK) cells [3]. However, IL-18 has been shown to accumulate in cancer patients, and increased serum IL-18 levels in cancer patients have been associated with tumor progression and a worse prognosis [4–6]. In one study, increased IL-18 levels were detected in serum and tumor tissues of patients with PC, and a higher tissue level of IL-18 correlated with increased metastasis and shorter survival [5].

Regulatory B cells (Bregs) have been shown to contribute to the maintenance of immune tolerance [7–9]. Bregs have been reported to produce either IL-10 or overlapping surface markers with diverse functions [10]. The following Bregs have been shown to suppress proinflammatory responses: IL-10–producing CD24hiCD38hi B cells, CD24hiCD27^+^ B cells (B10), CD38^+^CD1d^+^IgM^+^CD147^+^GrB^+^ B cells, CD27intCD38hi plasmablasts, and CD19^+^TIM1^+^ B cells [11]. Notably, within these different B-cell subsets, less than 20% of the B cells induced immunosuppression by producing IL-10. In addition, Bregs affected immunosuppression by stimulating secretion of other cytokines, such as lymphotoxin, IL-35, and transforming growth factor-beta (TGF-β), and directly interacting with immune cells through membrane surface molecules. Regulatory T cells (Tregs), DCs, NK cells, and cytotoxic T cells (Tc) can also be regulated directly or indirectly by Bregs [11]. Amounting data show that abnormalities in the number and function of Bregs promote development of several cancers [12–17]. Here, we studied the regulation mechanism of Bregs in PC patients and its possible correlation with IL-18 signaling.

## RESULTS

### Levels of bregs are higher in PC patients

**Figure 1:**
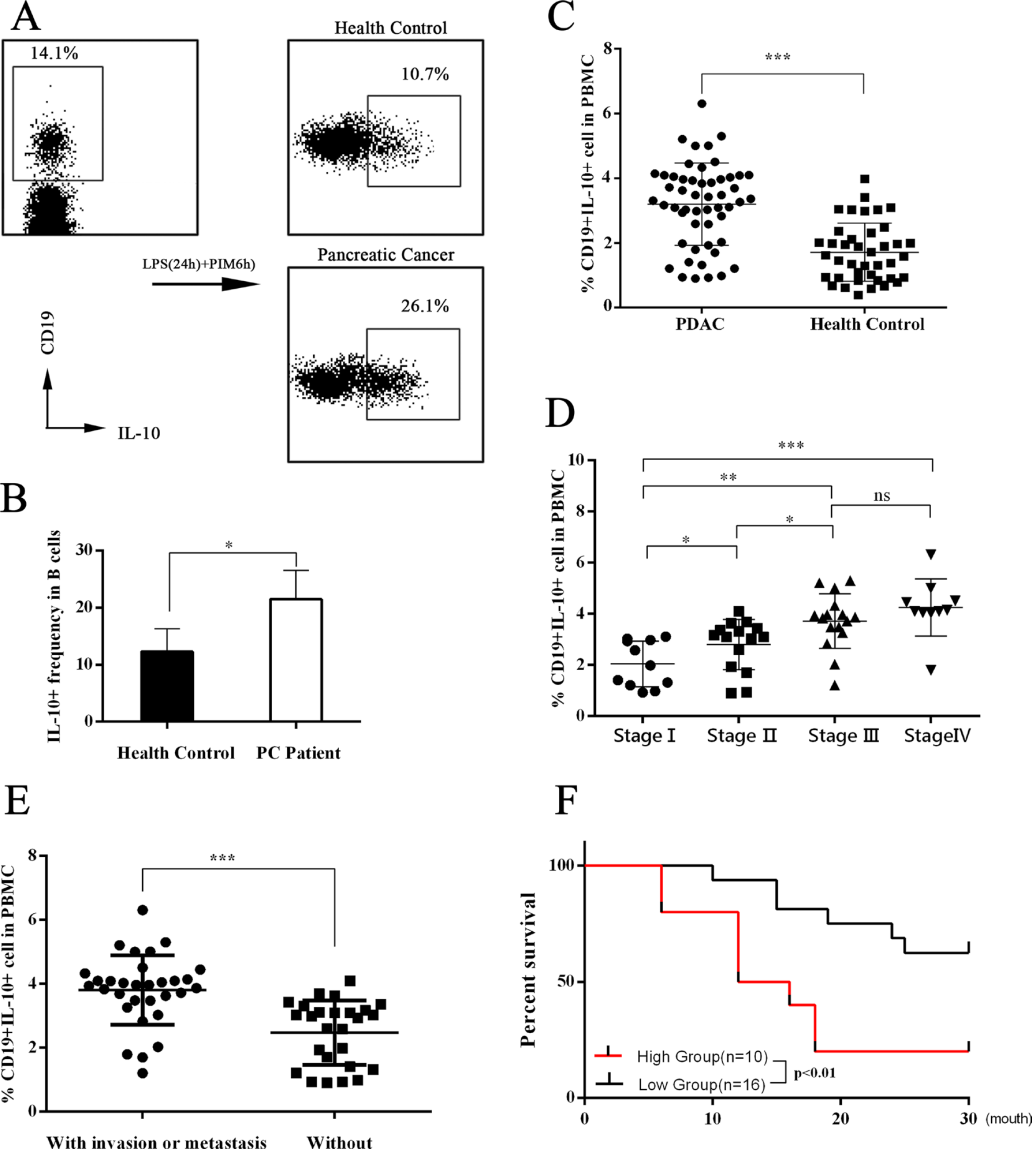
Assessment of IL-10 expression in peripheral blood B cells (**A**–**B**) The flow cytometry dot plots showed the IL-10 expression in cultured B cells. The CD19+ B cells were sorted from peripheral blood mononuclear cells (PBMCs) of both pancreatic cancer (PC) patients and healthy controls. Then CD19+ B cells were cultured *in vitro* with stimulation with LPS and PIB (last 6 hours). The presented flow cytometry data are from one experiment out of independent experiments. IL-10+ levels were 12.30 ± 1.789 (*n* = 5) in B cells of healthy controls and 21.50 ± 2.236 (*n* = 5) in B cells of PC patients. he summarized data are shown in (B). (**C**) The PBMCs were cultured *in vitro* with stimulation with LPS and PIB (last 6 hours). Then flow cytometry tests were performed. The summarized data are shown in (C). The Breg level was 3.199 ± 0.1762 (*n* = 52) in PC patients and 1.712 ± 0.1422 (*n* = 40) in healthy controls. (**D**) The 52 PC patients were divided into four groups according to TNM stage. The IL-10 expression levels were 2.043 ± 0.2709 (*n* = 11) in stage I patients, 2.798 ± 0.2542 (*n* = 15) in stage II patients, 3.716 ± 0.2680 (*n* = 16) in stage III patients, and 4.248 ± 0.3512 (*n* = 10) in stage IV patients. (**E**) The Breg level in PC patients with and without invasion and/or metastasis was analyzed. (**F**) According to the Breg level, stage I-II PC patients were divided into a high group and a low group, and the postoperative survival of the groups was analyzed. The summarized data are shown as means ± SEM. (ns = *P* > .05 and no significant difference; ^*^P < .05; ^**^P < .01; ^***^P < .001.).

Immune tolerance mediated by Tregs and Bregs is important for immune homeostasis. It has been suggested that dysregulation of Tregs and/or Bregs can be a causal factor in several cancers [10, 12–17]; however, the role of Breg dysregulation in PC remains unclear. To explore whether the Breg population was dysregulated in PC, we collected peripheral blood samples from PC patients and healthy subjects, sorted B cells by MACS, cultured B cells *in vitro*, and analyzed the IL-10^+^ B cells (Bregs) by flow cytometry. The results showed that the Breg level in PC patients was significantly higher than that of healthy controls (Figure 1A–1B). Furthermore, we found that the Breg level positively correlated with the tumor-node-metastasis (TNM) stage of PC (Figure 1C–1D). PC patients with invasion and/or metastasis had higher Breg levels (Figure 1E). Using the normal percentile method, we demonstrated a normal range of Bregs (0.31%–3.08%) in healthy subjects. Patients with stage I-II PC were divided into two groups according to the upper limit of normal range of Bregs: the high group and the low group. Then, a postoperative survival analysis was performed. The high group had worse overall survival (Figure 1F).

### IL-18 was overexpressed in plasma of PC patients, and IL-18R level was higher in IL-10^+^ B cells

**Figure 2:**
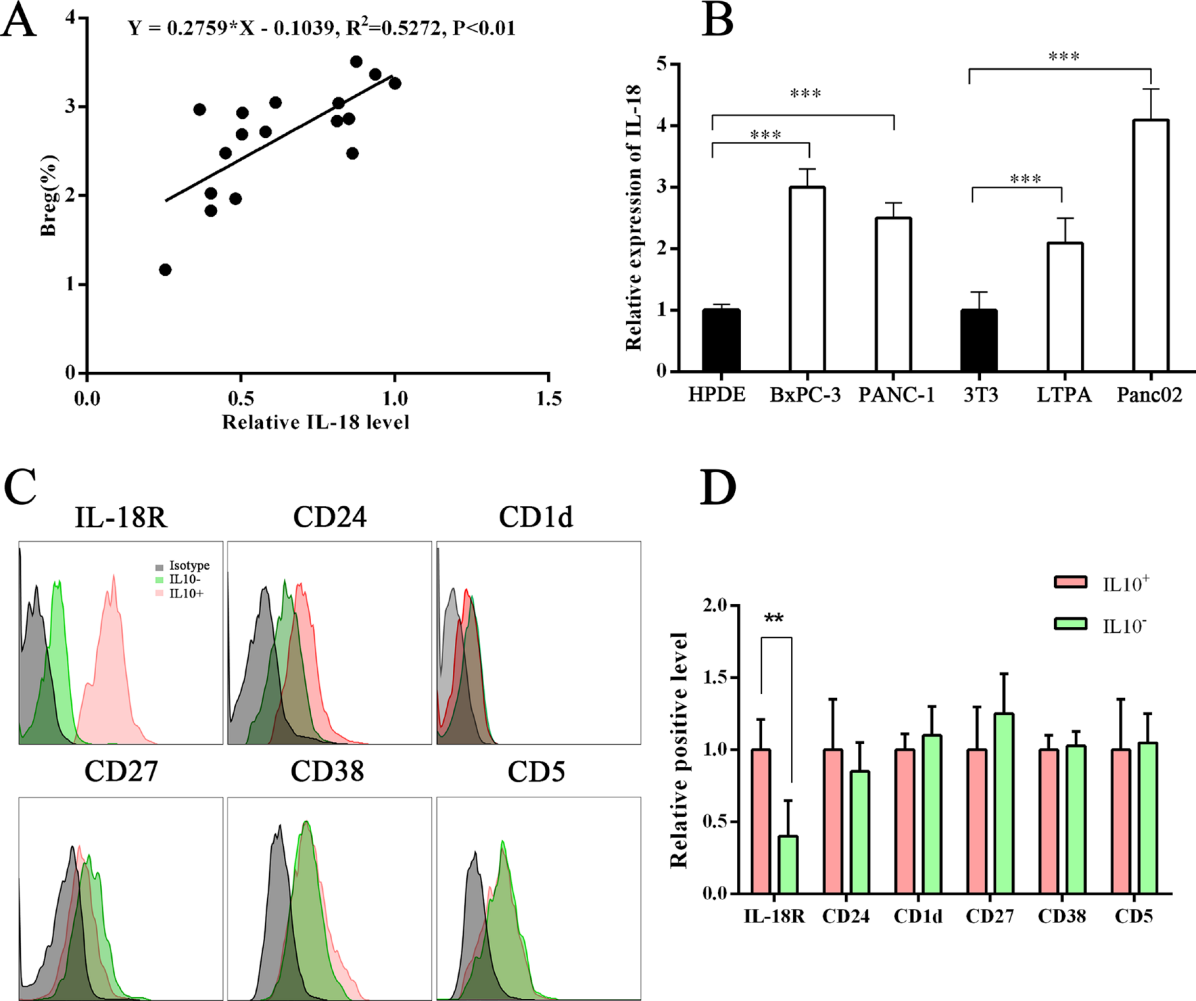
Correlation between Breg level and plasma IL-18 level (**A**) Graphs show a positive correlation between Breg level and plasma IL-18 level. Linear regression analysis showed R^2^ = 0.5272 and *P* < .01. (**B**) Graph showing the IL-18 level in the supernatant of normal cells and PC cells. (**C**–**D**) The Breg surface markers or IL-18R on B cells in PC patients were tested using flow cytometry. The IL-18R level was higher on IL-10+ B cells than on IL-10– B cells. The presented flow cytometry data are from one experiment out of independent experiments. (^**^P < .01; ^***^P < .001.).

IL-18 has both cancer-promoting and cancer-suppressing functions. Our previous study found that both plasma IL-18 and tissue IL-18 were upregulated in PC [5]. In this study, we analyzed the relationship between Breg and IL-18 levels and found that Breg level was positively correlated with IL-18 level (Figure 2A). We also analyzed IL-18R and several reported surface markers of Bregs. The IL-18R level was found to be higher in IL-10^+^ B cells (Bregs) than in IL-10– B cells (Figure 2B). These results indicate that the IL-18/IL-18R pathway is involved in Breg function.

### PC cell–derived IL-18 promoted B-cell proliferation and IL-10 production *in vitro*

**Figure 3:**
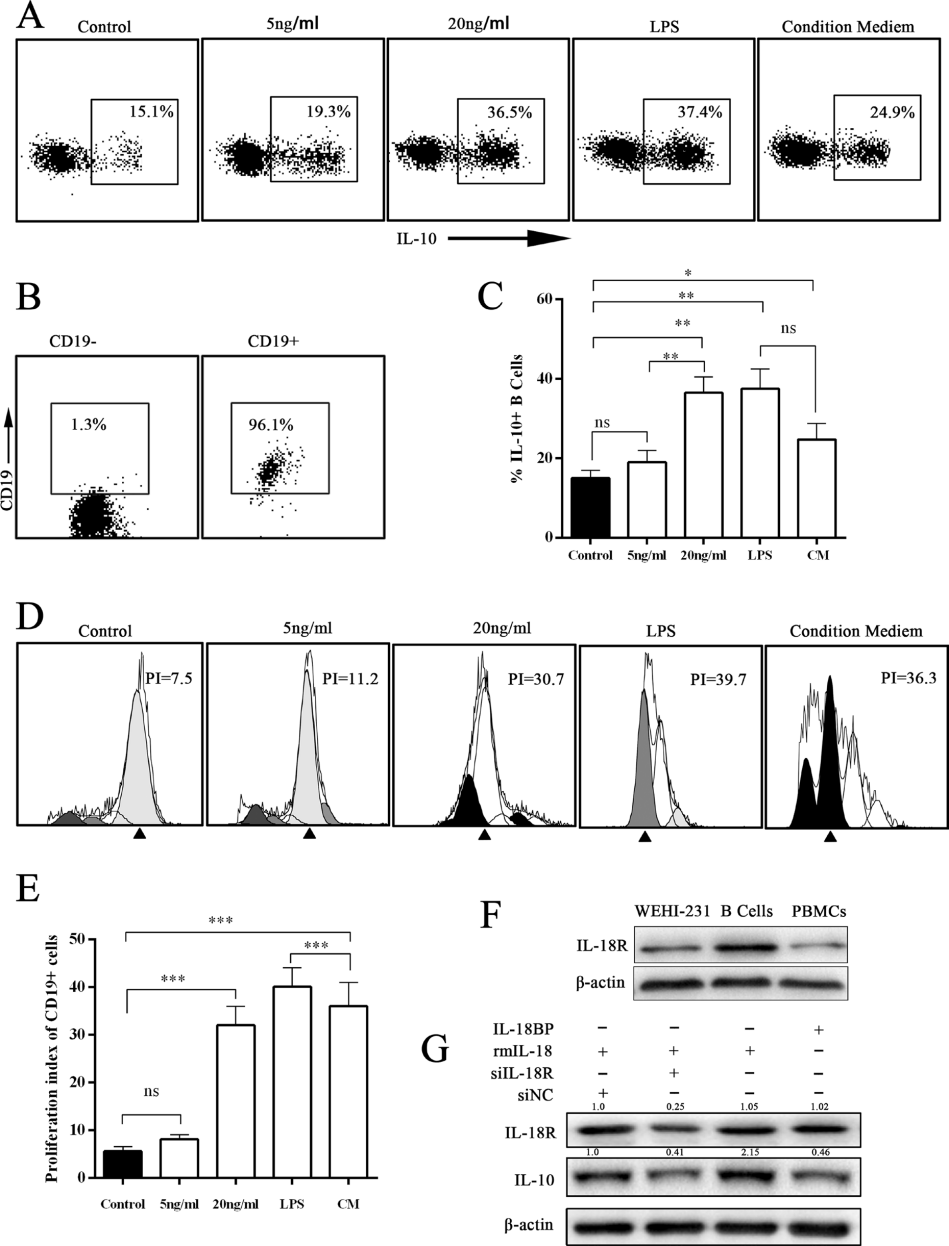
IL-18/IL-18R signal pathway induced IL-10 expression in B cells (**A**) The representative scatterplot figure show IL-10 expression in cultured B cells under different treatments with IL-18, LPS, or condition medium (CM) for 24 hours (PIB for last 6 hours). Then IL-10 expression was assayed by flow cytometry. (**B**) The flow cytometry assay of magnetic bead separation. (**C**) The summarized data of panel A are shown. (**D**) The CFSE assay was performed to analyze the proliferation of cultured B cells. (**E**) The summarized data of panel D are shown. (**F**) The expression level of IL-18R was analyzed by Western blot (WB). The presented data are from one experiment out of independent experiments. (**G**) The murine immature B-cell line WEHI-231 was stimulated with siIL-18R, IL-18, or IL-18BP. The expression level of IL-10 was analyzed by WB. (ns = *P* > .05 and no significant difference; ^*^P < .05; ^**^P < .01; ^***^P < .001.).

IL-18, an IL-1–related cytokine, can be produced by both immune cells and tumor cells [5, 6]. A previous study found that gut microbiota–derived IL-1β and IL-6 induced naïve B cells to differentiate into Bregs *in vivo* and *in vitro* [18]. We wondered whether IL-18 derived from PC cells had the same effect. First, we determined that the IL-18 level was significantly higher in PC cell culture supernatant by enzyme-linked immunosorbent assay (ELISA) (Figure 2C). Next, the B cells sorted from wild C57BL/J mouse peripheral blood were cultured under stimulation with different concentrations of rmIL-18 or condition medium. The results showed that both IL-18 and condition medium promoted IL-10 expression in B cells (Figure 3A–3C). In addition, the CFSE test revealed that both IL-18 and condition medium resulted in B-cell proliferation (Figure 3D–3E). Finally, in WEHI-231, a mouse B lymphocyte line, rmIL-18 promoted IL-10 production, which was interrupted by the natural IL-18 inhibitor, IL-18BP, or siIL-18R (Figure 3F–3G). These results indicate that IL-18 is a Breg inducer because it promotes proliferation and IL-10 expression in B cells.

### PC cell–derived IL-18 promoted immunosuppression *in vivo*

**Figure 4:**
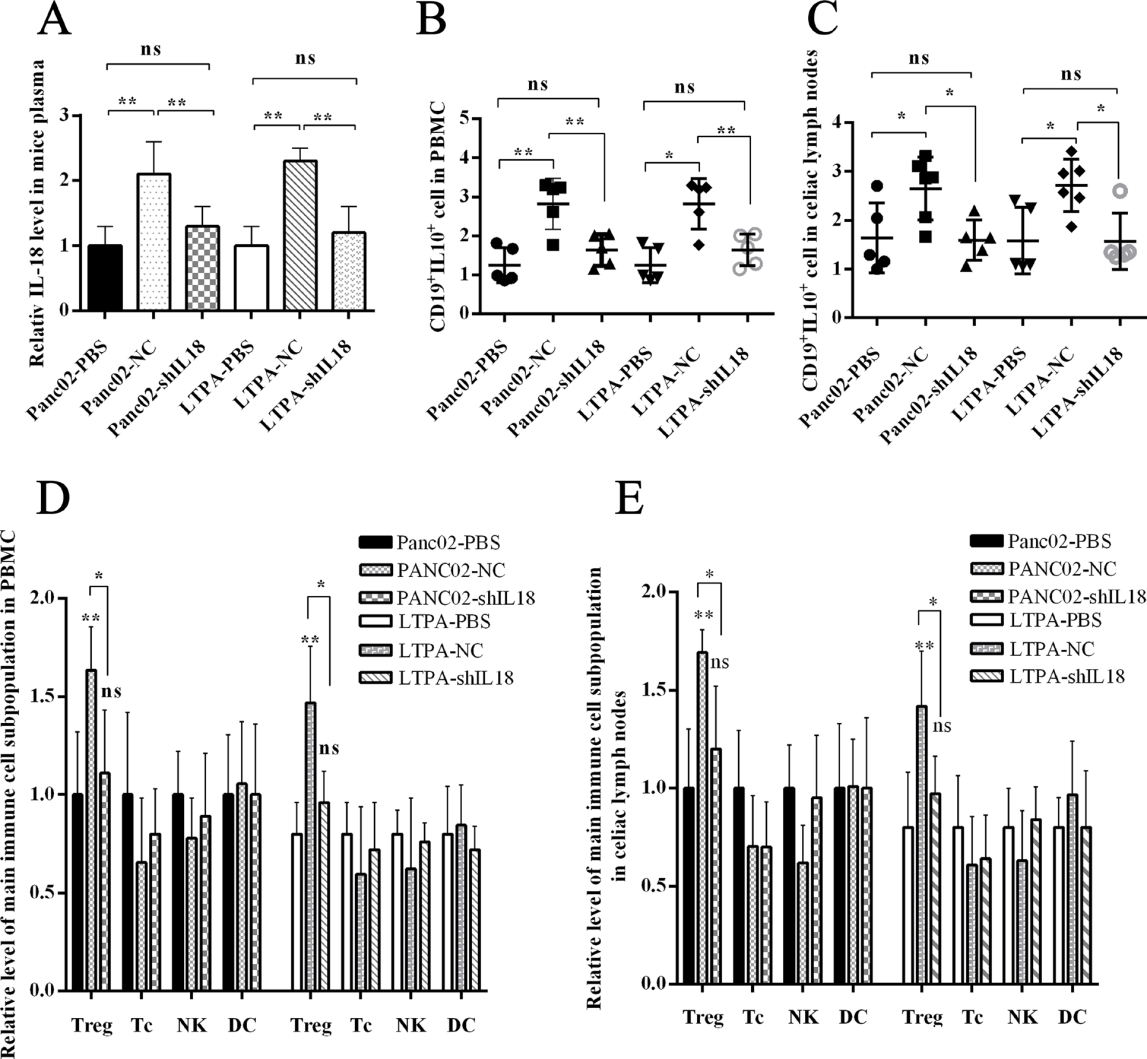
PC cell–derived IL-18 promoted immune tolerance *in vivo* The wild C57BL/6J mice were injected intraperitoneally with the murine pancreatic cancer cell lines Panc02 or LTPA. (**A**) IL-18 levels in the plasma of C57 mice were analyzed by ELISA. (**B** and **C**) The graphs show the Breg levels in PBMCs and celiac lymph nodes. (**D** and **E**) The graphs show the frequency of the main immune cell subpopulations in PBMC and celiac lymph nodes. (ns = *P* > .05 and no significant difference; ^*^P < .05; ^**^P < .01; ^***^P < .001.).

We aimed to determine whether IL-18–induced B cells were immunoregulatory B cells (Bregs) *in vivo*. We intraperitoneally injected wild C57BL/6J mice with murine PC cell line Panc02 cells or LTPA cells. After 1 week, the immune cell subsets were analyzed by flow cytometer. We found that the Breg and Treg levels in peripheral blood and celiac lymph nodes were significantly higher in the Panc02-NC/LTPA-NC interference group than the Panc02-shIL18/LTPA-shIL18 interference group or control group. Similarly, the plasma IL-18 level was higher in the Panc02-NC/LTPA-NC interference group than in the Panc02-shIL18/LTPA-shIL18 interference group or control group (Figure 4A–4E). These *in vivo*/*in vitro* results indicate that PC cells possibly gained immune tolerance through IL-18 production, which promoted the generation of immunosuppressive cells, such as Bregs and Tregs.

### IL-18 stimulation resulted in increased PD-L1 expression in B cells

**Figure 5:**
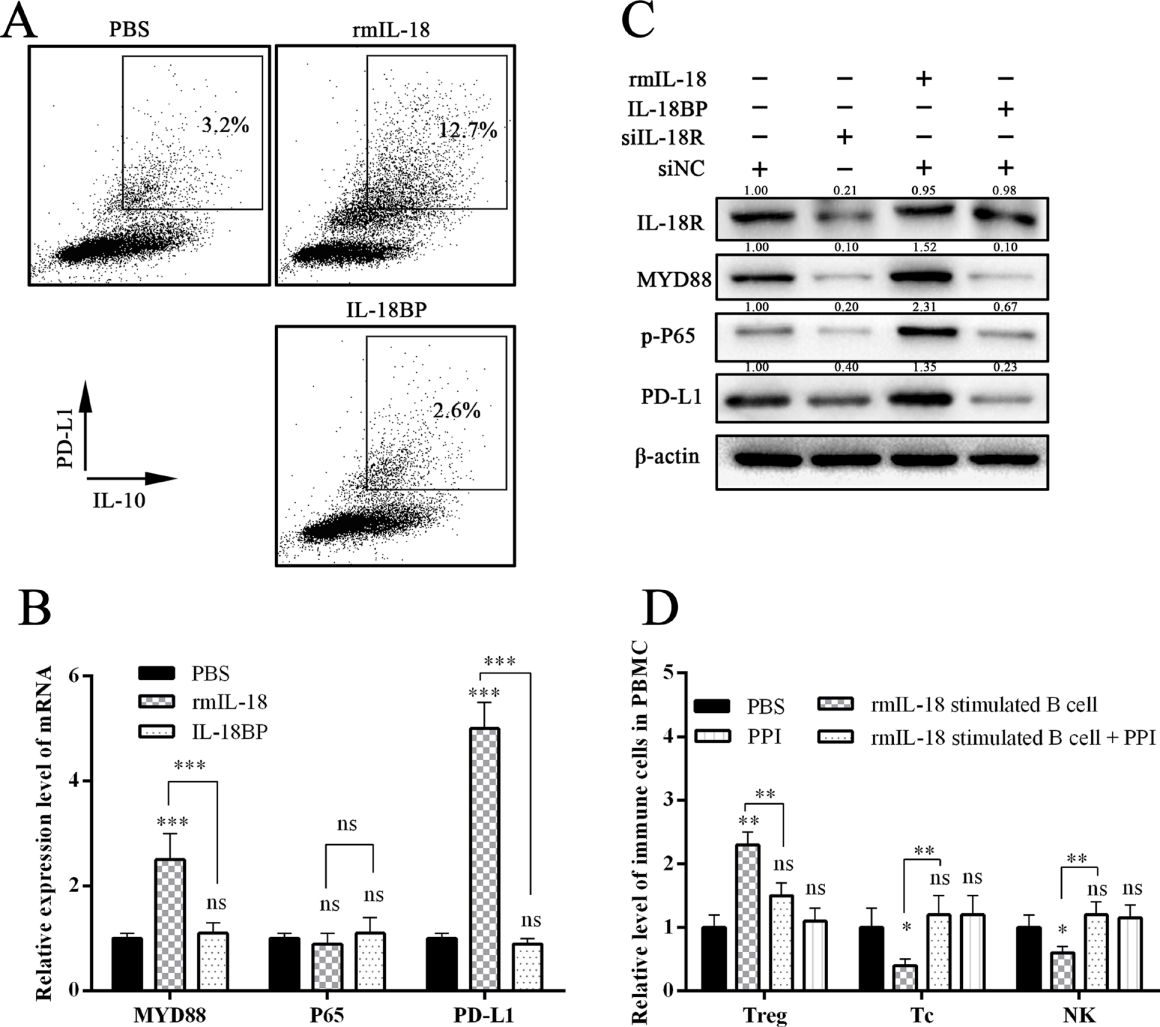
IL-18 promoted PD-1 expression in B cells (**A**) The flow cytometry dot plots showed the IL-10 and PD-1 levels in B cells after stimulation with PBS, rmIL-18, or IL-18BP. (**B** and **C**) The graphs show the expression levels of key genes in the downstream of the IL-18/IL-18R pathway. (**D**) The wild C57 mice were injected via tail vein with the stimulated B cells, which were co-cultured with or without PD-1/PD-L1 inhibitor (PPI). One week later, the immune subsets in peripheral blood were tested. The summarized data are shown. (ns = *P* > .05 and no significant difference; ^*^P < .05; ^**^P < .01; ^***^P < .001.).

Recently, it has been suggested that tumor-infiltrated B cells develop immunosuppressive properties via enhanced expression of TGF-β, PD-L1, CD86, and IL-10 [19]. Because PC cell–derived IL-18 promoted Breg generation, we wondered whether IL-18–induced Bregs had increased expression of these proteins. We found that WEHI-231 expressed more PD-L1 under rmIL-18 stimulation than with phosphate-buffered saline (PBS) or IL-18BP treatment, which inhibited the IL-18/IL-18R pathway. In addition, the level of PD-L1^+^IL-10^+^ B cells was higher in the rmIL-18 stimulation group than the other groups (Figure 5A). We also analyzed the key downstream gene of IL-18R in WEHI-231 and found that rmIL-18 upregulated MYD88 and PD-L1 levels and promoted phosphorylation of P65 (Figure 5B–5C). These results indicate that PD-L1 could be another marker of IL-18–induced Bregs.

### PD-L1/PD-1 inhibitor treatment inhibited IL-18–induced immunosuppression *in vivo* and *in vitro*

**Figure 6:**
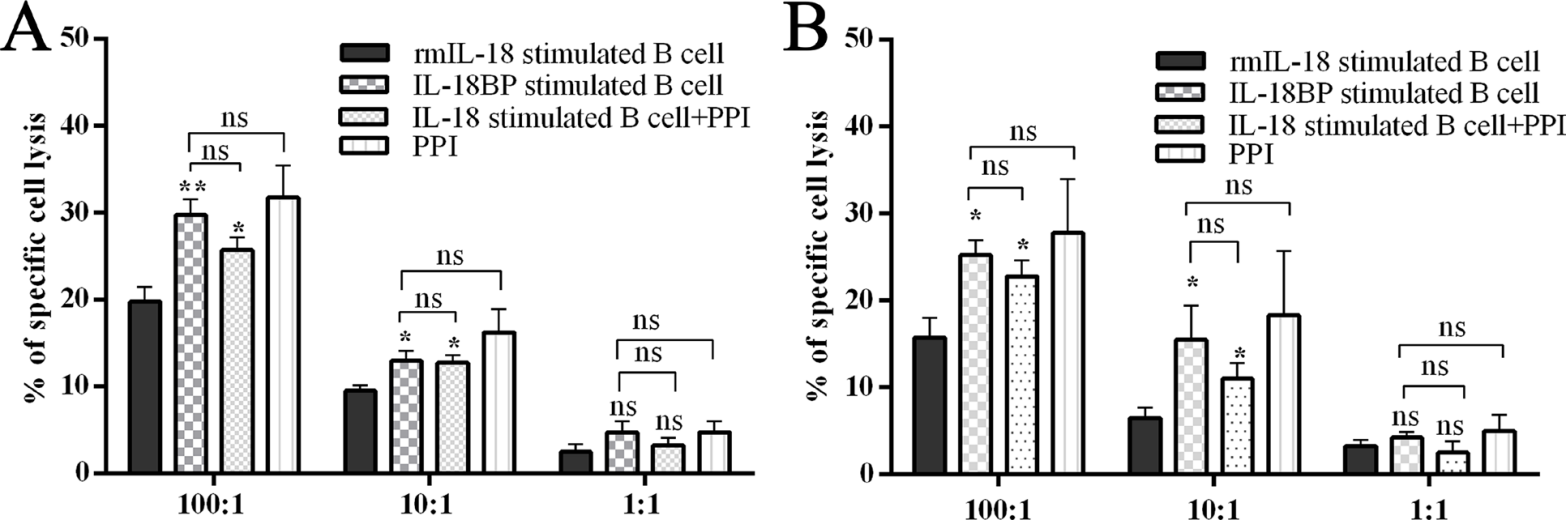
Breg capacity could be interrupted by inhibiting the IL-18/IL-18R pathway or PD-1/PD-L1 pathway The MASC isolated effector cells, murine NK cells, and CD8a+ Tc cells were co-cultured with B cells, which were stimulated with or without rmIL-18 in advance and with or without PPI. Then effector cells were co-cultured with target cells at ratio of 100:1, 10:1, or 1:1. Twenty hours later, the LDH level in culture supernatant was tested as an indicator of antibody-dependent cellular cytotoxicity (ADCC) assay. The results are shown in panels (**A** and **B**). (ns = *P* > .05 and no significant difference; ^*^P < .05; ^**^P < .01; ^***^P < .001.).

Recently, anti–PD-L1/PD-1 immune therapy has been used to treat advanced human cancers. Because we showed that PD-L1 is a marker of PC-induced Bregs, we wondered whether blocking the PD-L1/PD-1 pathway would disturb the Breg-mediated tumor immune tolerance. We co-cultured the sorted mice NK cells and Tc cells with rmIL-18/IL-18BP–stimulated B cells and then added, or did not add, a PD-L1/PD-1 inhibitor (PPI). The antibody-dependent cellular cytotoxicity (ADCC) assay was then performed. The results revealed that rmIL-18–stimulated B cells significantly impaired the ADCC of Tc cells and NK cells; this effect was reversed by PPI treatment (Figure 6A–6B). We injected the wild C57 mice with the stimulated B cells via tail vein and tested the immune subsets of peripheral blood 1 week later. Treg levels were upregulated and Tc/NK levels were downregulated in the rmIL-18 stimulation group. However, PPI treatment inhibited these effects (Figure 5D). These results indicate that the PD-L1/PD-1 pathway is important in IL-18–induced immunosuppression *in vivo* and *in vitro* and that PD-L1 should be a good marker of Bregs in PC.

### The combination of IL-18BP and PD-L1/PD-1 inhibitors suppressed PC growth and metastasis

**Figure 7:**
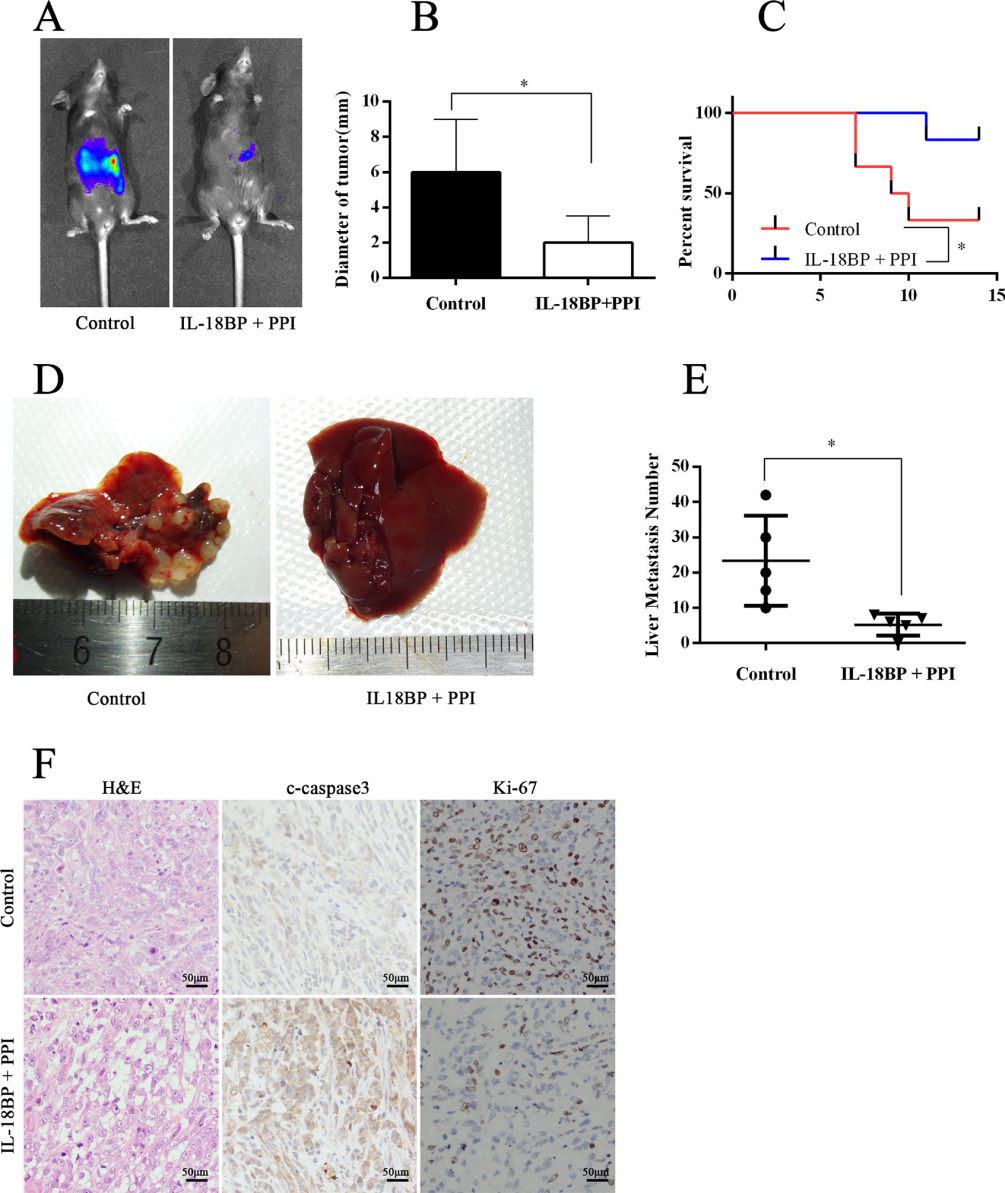
The combination of IL-18BP and PD-L1/PD-1 inhibitors suppressed PC cell growth and metastasis The PC orthotopic implantation model was established in C57BL/J mice with Panc02-lucifer cells. One week later, the mice received intraperitoneal injections of PBS or a combination of IL-18BP and PPI. (**A**) The graph showed the fluorescence intensity of orthotopic tumor at the sixth week. The presented data were from one experiment out of independent experiments. (**B**) The summarized data of tumor diameter. (**C**) The survival analysis. (**D**) The liver gross observation. (**E**) Hematoxylin and eosin (H&E) staining of livers revealed metastasis foci. The summarized data are shown. (**F**) The tumors were formalin fixed and paraffin embedded and H&E stained or histochemically analyzed. The results are shown. The presented data are from one experiment out of independent experiments. (ns = *P* > .05 and no significant difference; ^*^P < .05; ^**^P < .01; ^***^P < .001.).

Because tumor cell–derived IL-18 and the PD-L1/PD-1 pathway are important in PC cell–induced immune tolerance, targeting these factors may constitute a new immunotherapeutic regimen for PC. We established a PC orthotopic implantation model in C57BL/J mice with Panc02-lucifer [20]. From the second week on, mice received intraperitoneal injections with PBS or IL-18BP+PPI once per week. At week 8, we tested the fluorescence intensity of orthotopic tumors. Some mice were executed with anesthesia, whereas others were reserved for survival observation. The results showed that mice treated with IL-18BP+PPI had smaller tumor size, healthier livers, and fewer liver metastases than mice injected with PBS (Figure 7A–7B and 7D–7E). The liver metastasis foci under microscopic observation revealed the same trend. Meanwhile, the apoptosis and proliferation status in the orthotopic implantation tumor was measured and it was found that the tumor tissue showed more apoptosis and decreased proliferation in mice treated with IL-18BP+PPI (Figure 7F). Mice treated with IL-18BP+PPI also showed a higher survival rate and lower incidence of cachexia or ascites (Figure 7C).

## DISCUSSION

PC is predicted to be the second leading cause of cancer deaths by 2030, given its 5-year survival rate of 8%. Approximately 80% of PC patients are diagnosed with disease that is too advanced for surgery. Moreover, PC can be resistant to chemoradiation [1]. Thus, more effective therapies need to be established. As a result of the greater understanding of the PC immune microenvironment over the past decade, it has been determined that most clinically evident cancers have evolved to evade immune surveillance [21]. According to the “cancer immunoediting” hypothesis, the interaction between the immune surveillance system and cancer cells occurs in three phases: the elimination phase, the equilibrium phase, and the escape phase. Of note, in the equilibrium phase, the adaptive immune system prevents tumor outgrowth but fails to eliminate the tumor, which establishes the immune-mediated evolution of a tumor. Cancer cells may evolve during this phase by reducing immunogenicity and/or promoting immune tolerance [22]. Bregs exist as a subset of immune suppress cells, and dysregulation of frequency or property of Bregs is involved in the tumorigenesis of several cancers, including tongue squamous cell carcinoma, prostate cancer, lung cancer, breast cancer, and liver cancer [10, 12–17]. In this study, we found that higher Breg levels existed in the peripheral blood of PC patients than in healthy controls and that Breg levels were positively correlated with TNM stage of PC. In addition, PC patients with invasion and/or metastasis had higher Breg levels, and higher Breg levels were associated with worse overall survival (Figure 1). Using Panc02/LTPA abdominal injection murine models, we found that the murine PC cell lines Panc02 and LTPA upregulated immunosuppressive Bregs and Tregs in wild mice (Figure 4). These results demonstrate that PC cells induce immune tolerance, which is associated with increased immunoregulatory cells, such as Bregs and Tregs. A variety of publications have reported that Tregs are a substream of Bregs [9, 12, 14, 17, 23], which indicates that increased Bregs are likely more important for tumor-induced immune tolerance in PC.

IL-18, as a costimulatory factor, is involved in IL-12–mediated IFN-γ production by T-helper cells, since most peripheral CD4+ T cells express IL-18R [24]. IL-18 is predominantly expressed by myeloid and epithelial cells. As a precursor protein, IL-18 requires protease-mediated cleavage (commonly by caspase-1) to be biologically active [25, 26]. Although IL-18 is perceived as an anticancer cytokine, high concentrations of IL-18 have been associated with advanced tumor stages in patients with seven tumor types (pancreatic cancer, esophageal squamous cell carcinoma, breast cancer, hepatocellular carcinoma, lung cancer, renal cell carcinoma, multiple myeloma, and oral cavity cancer) [5, 6]. Tumor-derived IL-18 was found to promote Kit+ NK cell transformation and mediate immunoablative functions in NK cell–controlled cancers [27]. In a previous study, we found that IL-18 levels were increased in plasma and tissue [5]. Furthermore, we found that plasma IL-18 levels were positively correlated with peripheral blood Breg levels in PC patients (Figure 1A) and that IL-18R was a surface marker of Bregs (Figure 2B). In addition, tumor-derived IL-18 or rIL-18 enhanced B-cell proliferation and IL-10 production *in vitro* and *in vivo* (Figure 3 and 4). These results indicate that the IL-18/IL-18R pathway is important in PC-induced immune tolerance.

PD-L1 (B7-H1), one ligand of PD-1, is expressed to varying degrees in epithelial and hematopoietic cell populations [28]. PD-L1/PD-1 signaling is known to attenuate signaling from the T-cell antigen receptor (TCR) and inhibit the population expansion, cytokine production, and cytolytic function of T cells [29]. Studies of PD-L1/PD-1 have suggested its importance in regulating humoral immune responses. Moreover, surface-expressed PD-L1 was also recognized as an important feature of Bregs, which play a critical role in regulating humoral immunity mediated by CD4+CXCR5+PD-1+ follicular helper T cells [30]. In the present study, we found that IL-18 upregulated PD-L1 expression (Figure 5A-5C) and that the frequency of IL10+PD-L1+ B cells increased significantly after rmIL-18 stimulation in cultured B cells. In addition, treatment with a PPI attenuated the ADCC repression of rmIL-18–induced Bregs *in vitro* (Figure 6). Moreover, combined treatment with IL-18BP and a PPI significantly repressed tumor growth and metastasis in the PC orthotopic implantation model (Figure 7). The PD-L1/PD-1 pathway represents another important part of PC-induced immune tolerance.

In conclusion, IL-18 promoted the differentiation of naïve B cells into CD19+IL10+ Bregs, which further enhanced PC immune tolerance. IL-18/IL-18R and PD-L1/PD-1 may be promising therapeutic targets in patients with PC.

## MATERIALS AND METHODS

### Ethics statement

This study was approved by the Human Ethic Committee and Animal Care Committee of Tongji Hospital, Wuhan, China. Written informed consent was obtained from each subject. All of the experiments involving humans and animals were carried out according to the approved guidelines.

### Patients and samples

Blood samples were obtained between January 2013 and October 2016 from 52 PC patients and 40 healthy subjects. The peripheral blood was collected via ulnar vein puncture. The plasma IL-18 level was analyzed by ELISA kit (Boster, China) following the manufacturer’s guidance. Peripheral blood mononuclear cells (PBMCs) were isolated from the peripheral blood for flow cytometry. For the magnetic cell sorting of CD19+ B lymphocytes (Miltenyi, Germany), a total of 10 mL of peripheral blood was collected for detection.

### Reagents and mice

The flow cytometry antibodies used in this study included: (1) the reagents from BD Pharmingen (United States), such as human APC/PE anti-CD19, human PE/APC anti-IL10, human PE anti-IL18R, human PE anti-PD-L1, mouse PE anti-CD4, mouse APC anti-CD3, mouse PE anti-PD-L1, mouse PE anti-IL10, mouse FITC anti-Dx5, mouse APC-CY7 anti-NK1.1, and others; (2) reagents from eBioscience (United States), such as mouse APC anti-CD3 and others; and (3) reagents from Miltenyi, such as the mouse Tregs detection kits, the human Tregs detection kit, mouse-cell isolation kit, human CD19+ B cell isolation kit, mouse NK Cell Isolation Kit II, mouse CD8a+ T Cell Isolation Kit, and others. In addition, the recombinant human/mouse IL-18 and human/mouse IL-18BP were purchased from R&D Systems (United States), and the PD-L1/PD-1 inhibitor was obtained from Selleck Chemicals (United States).

The wild C57BL/J mice used in this study were female and approximately 6 to 8 weeks old. For the intraperitoneal injection, 100 μL of Panc02 or LTPA cell suspension at a concentration of 2 × 107 was used. One week later, the mice were sacrificed by anesthesia. The peripheral blood and the celiac lymph nodes were collected. For the PC orthotopic implantation model, 50 μL of Panc02-lucifer cell suspension in a concentration of 2 × 107 were injected in the mouse pancreas after anesthesia and laparotomy. Then the abdomen was closed layer by layer. After 8 weeks, the mice were injected with 150 mg/kg of D-luciferin via tail vein after anesthesia. Then the mice were placed in the bioluminescent imaging system (IVIS Lumina XR; Caliper, United States), and the white light and bioluminescence images were captured.

### Cell culture

The murine PC cell line Pan02, the murine B immature lymphocyte WEHI-231, the murine embryonic fibroblasts cell line 3T3, and the human PC cell PANC-1 were cultured with DMEM medium supplemented with 10% FBS, 100 U/mL of penicillin, and 0.1 mg/mL of streptomycin. The murine PC cell line LTPA, the human PC cell line BxPC-3, and normal pancreatic ductal epithelial cell line HPDE were cultured with RPMI1640 medium supplemented with 10% FBS, 100 U/mL of penicillin, and 0.1 mg/mL of streptomycin. The condition medium was a mixture of fresh RPMI1640 medium supplemented with 10% FBS and pancreatic cancer cell culture supernatant at a ratio of 1:1.

### Co-culture and CFSE (proliferation assay)

For the CFSE analysis, CD19+ cells were stained with Cell Trace CFSE (Invitrogen, United States) in accordance with the manufacturer’s guidance. For the co-culture, CD19+ cells were cultured in RPMI-1640 with 10% FBS, 100 U/mL of penicillin, and 100 U/mL of streptomycin or condition medium. Next, cells were stimulated with PIB (PMA, ionomycin, BFA) for 0, 48, and 96 hours for flow cytometric analysis.

### Flow cytometry

For cell surface staining, cells were incubated with flow cytometry antibodies or isotype IgG for 30 minutes at 4°C. After washing with PBS, the cells were analyzed with flow cytometer.

The intracellular staining for IL-10 was performed after stimulation. Briefly, freshly acquired CD19+ B cells were cultured in RPMI 1640 medium supplemented with 10% FBS, 100 U/mL of penicillin, 100 U/mL of streptomycin, and phorbol 12-myristate13-acetate (PMA) (50 ng/mL) for 48 hours and ionomycin (1000 ng/mL, both Sigma-Aldrich, United States) and brefeldin A (10 ng/mL, BFA, BD Biosciences, United States) for another 6 hours. Then the PBMCs (suspended in PBS with 5% FBS and 0.1% NaN3) were incubated with surface marker antibodies, such as anti-CD19 antibody. After fixation and permeabilization (Biotech, China), cells were stained with intracellular marker antibodies, such as anti–IL-10 antibody. After washing with PBS, the cells were analyzed with flow cytometer.

### Antibody-dependent cellular cytotoxicity (ADCC)

ADCC assays were performed at an effector-to-target (E:T) ratio of 100:1, 10:1, or 1:1. The effector cells, NK cells and CD8a+ T cells, were isolated from single-cell suspensions of mouse spleen using the mouse NK Cell Isolation Kit II (Miltenyi) and the mouse CD8a+ T Cell Isolation Kit (Miltenyi). At first, the effector cells were stimulated with rmIL-18–stimulated B cells, IL-18BP–stimulated B cells, a combination of IL-18–stimulated B cells and PPI, or PPI only. Then the effector cells were co-cultured with target cells, 10,000 cells/well, in a U bottom 96-well plate. Twenty hours later, the supernatant was collected for lactate dehydrogenase (LDH) release measurements using LDH Cytotoxicity Assay Kit (Cayman, United States).

### Real-time quantitative reverse transcriptase polymerase chain reaction (RT-qPCR)

RNA was extracted from sorted cells with Trizol and reverse transcribed into cDNA with the Reverse Transcription System (Takara, Japan). Real-time RT-qPCR was performed on an ABI 7900 System with PrimeScript RT Master Mix (Takara).

### Statistical analysis

Results for continuous variables are presented as means ± standard deviation (SD) unless stated otherwise. Treatment groups were compared with independent sample by *t* tests. Pairwise multiple comparisons used one-way analysis of variance (two-sided). *P* < .05 was considered statistically significant. All analysis was performed using SPSS v.17.0 software (SPSS Inc., United States).

## Abbreviations

IL-18BP: interleukin-18 binding protein
Tc cells: cytotoxic T cells
NK cells: natural killer cells
MASC: magnetic activated cell sorting
MYD88: myeloid differentiation primary response gene 88
ADCC: The antibody-dependent cellular cytotoxicity
